# What are the Characteristics of an Excellent Review of Scientific Articles?

**DOI:** 10.5935/abc.20180032

**Published:** 2018-02

**Authors:** Carlos Eduardo Rochitte, Claudio Tinoco Mesquita

**Affiliations:** 1 Instituto do Coração do Hospital das Clínicas da FMUSP (InCor - HCFMUSP), São Paulo, SP. - Brazil; 2 Hospital do Coração (HCOR), São Paulo, SP. - Brazil; 3 Universidade Federal Fluminense (UFF), Niterói, RJ. - Brazil; 4 Hospital Pró-Cardíaco, Rio de Janeiro, RJ - Brazil

**Keywords:** Authorship and Co-Authorship in Scientific Publications, Scientific and Technical Publications, Peer Review, Peer Review, Research, Journal Impact Factor

The selection by a scientific journal's editor-in-chief and associate editors of a manuscript for publishing is mainly, although not exclusively, based on the opinion of the manuscript's reviewers. This process is known as peer review and consists on the manuscript's assessment by experts in the area, who judge the scientific merit of the manuscript submitted to the journal. This process is expected to accept the better science for publishing, while refusing that of lower merit. Other standards and rules followed by editors of international journals contribute to improve the scientific quality of the journals.^1^ One of the most important contributions of peer review is the refinement of the manuscript regarding its clarity and content. To optimize the reviewer's contribution in this process, understanding the characteristics involved is necessary. 

In the peer-review system, it is crucial that the reviewer's scientific opinion be transmitted to the editors in a clear and focused way regarding the essential aspects for decision making. This information is conveyed through writing by the reviewer in the review system of a given journal. Dealing with online article submission and review systems is challenging, and most of such systems are neither intuitive nor easy to use. However, this editorial will not focus on such difficulties, which can usually be overcome with the support of assistant editors and an efficient editorial office, which we fortunately have for the Brazilian Society of Cardiology (SBC) journals: the *Arquivos Brasileiros de Cardiologia* and the *International Journal of Cardiovascular Science*. We will address the specific topics that should be clearly indicated by the reviewers to allow the editors to make their best decision possible. In addition to local specific suggestions that the SBC journals' editors consider important, we add recommendations of other editors for an excellent-quality review.^2,3^

An excellent review requires time and effort of the reviewer, in addition to a non-trivial work of checking the literature in the manuscript's specific area. That time tends to decrease as the reviewer gains more experience, but, on average, it ranges from 2 to 3 hours. The reviewer is rewarded with the knowledge and updated view of the specific area, in addition to the possibility of influencing the text that will be read by the scientific cardiovascular community. An excellent review will play a crucial role in the manuscript's acceptance or rejection, as well as significantly improve the manuscript's quality. It is a great opportunity for the reviewer not only to participate in the dissemination of innovation and new knowledge, but to directly influence it, in addition to being aware, prior to other colleagues, of the innovations that are in the pipeline, that is, in the publishing process. Usually, our reviewers are chosen based on their capability and technical knowledge in cardiovascular science and their history of publishing in that specific field, which make them highly trained in article editing, often qualifying them as excellent reviewers. However, the process of article selection usually requires specific responses focused in certain aspects of the manuscript that can pass unnoticed by the reviewer. In addition, different journals can differ in the way reviewers and editors communicate. Many reviewers never receive any formal guidance on what editors consider essential in reviews. This document will provide the reviewers with the information the *Arquivos Brasileiros de Cardiologia* and *International Journal of Cardiovascular Science* editors would like to find in an excellent review for their journals.

The scientific reviewers are invited to represent the journals in selecting articles of high scientific quality for publishing. The reviewers should protect our journals from articles with evident flaws or with methodological errors, inappropriate analysis or conclusions. In that aspect, the reviewers act as judges. In addition, they are expected to act as consultants to the authors to improve the article. Another characteristic of the process is that almost all articles that undergo peer review, whether accepted or not for publishing, end up improved.

Many reviews begin with a short summary of the manuscript. Although the editors have already read the manuscript, this summary provides them with the perspective of the reviewer, an expert in the area. Thus, the manuscript's summary, although not mandatory, is extremely useful for the editors and highly recommendable.

The essence of a review is the manuscript's assessment and how it will serve the scientific process. The reviewer should ask himself the following questions: Is there a rationale for the study's objectives? How important is the hypothesis being tested? The term 'important' here can have several meanings, depending on the subjective view of each reviewer, but a point considered critical is whether the hypothesis is original and has not been tested before in the literature. The famous gap in the literature is what we search in a manuscript to justify its publication. Metaphorically speaking, we look for a hole in the cardiovascular science wall to put a brick in it. Or, more directly speaking: Is it new? Is it true? Does anybody care about it? Or: Is the manuscript original, precise, valid and relevant?

Continuing with the technical questions on the manuscript being reviewed, reviewers should ask: Are the methods for data collection and analysis appropriate and accurate? Are the results significant for the area? Can the conclusions be supported by the results? We suggest such questions be divided into two types: general and specific comments. The general comments are the most important ones and should comprise the manuscript's positive and negative general aspects. For example, if there is an important methodological flaw or if the sample size is insufficient, or if originality is a strength. Those aspects should be part of the general comments. The specific comments comprise grammar or sentence corrections and suggestions of change in tables and figures, which are formal aspects to be fixed, and the reviewer should indicate their respective page and paragraph.

It is worth noting how frequent such data lack in the reviewer's comments, leaving the interpretation for the editors. Would that lack of information indicate that the article is suitable for publication?

The best reviews compare the manuscript with the current literature in the specific area, in addition to providing the references that support the reviewer's opinions, especially regarding the manuscript's originality. Quite often, editors must judge a manuscript based on different opinions from different reviewers. Very likely, the opinion supported by the literature will prevail.

A common mistake in our editing management system is when reviewers repeat the comments to authors in the space reserved for comments to editors. This space is intended for confidential comments, where reviewers are free to justify directly why they accept or reject the manuscript, or even suggest its rejection or acceptance, justifying their decision. In that same space, reviewers can comment if the manuscript is suitable for our audience. Although this is a fundamental task of editors, the reviewer's opinion will be considered, and often the editors will follow the reviewer's opinion.

It is worth noting and emphasizing that, in the 'comments to authors' section, reviewers should never state whether the manuscript would or would not be accepted. The authors should only receive comments on specific scientific merits and suggestions for improvement. Nevertheless, despite the determinant role of the review in the fate of the manuscript, the final decision of acceptance or refusal is up to the editors, and, eventually, to the editor-in-chief. 

It is worth noting the practical fact that the review is undoubtedly a very individual process, to which there is no formal training, and, similarly to medicine, an art. Thus, the result of the scientific review is necessarily a mix of scientific merit and the reviewer's opinion. From the editors' viewpoint, reviewers must acknowledge that our journals, whose best impact factor is 1.18, will receive manuscripts with scientific limitations inherent in any submission, but possibly more evident in our cases. In this context, the reviewer should decide whether the manuscript, despite its limitations, deserves to be published or not, and communicate that clearly to the editors, in the 'confidential comments to editors' space. Excessive rigidity is not recommended at that point. Assess and reflect. Be neither aggressive nor rude. Be technical. Remember the large amount of effort the colleagues put into the task, from the project elaboration to the manuscript's final writing. In the next step, the reviewer should act as a consultant to authors, clearly indicating which changes should be made to provide the manuscript with quality for publishing.

Finally, be concise. Short and objective texts, and even a list of items of the changes suggested, are sufficient. Do not exceed one page of text with single line spacing. We do not recommend long reviews with endless lists of changes. Even the specific comments on shape and grammar, if frequent in the manuscript, can be summarized as only one suggestion of extensive grammar review. Our journals can use writing consultants in English and Portuguese. The same is valid for the statistical analysis, for which we count on a statistical appraiser and consultant for all manuscripts submitted.

The review of scientific articles and reviewers are extremely important for the scientific community in general and for the existence of the journals. Despite the increasing trend towards previous publishing in repositories prior to peer review to accelerate the dissemination of the results, peer review is fundamental for the reliability of an article in the scientific community. Thus, the review of scientific manuscripts is a huge responsibility of inestimable value, which leads editors to keep in mind the names of the high-quality reviewers. To confirm that value, we will go beyond the prizes for the most punctual reviewer, enhancing the awards and recognition in our scientific community for reviewers who perform best. Wait and see!

The following table summarizes our recommendations for reviewers.

We hope to have contributed to foster an efficient dialogue between reviewers and editors in the coming years, in addition to yielding an increasingly suitable selection of articles for publishing in our journals.

## Figures and Tables

**Table:** 

RECOMMENDATION FOR SCIENTIFIC REVIEWERS	
**STRUCTURE OF THE REVIEW**	
**COMMENTS TO AUTHORS**	
1. Manuscript summary from the reviewer's perspective	How the reviewer "sees" the article. Describe in your own words the objectives, methods and important findings. How does the article compare in the literature?
2. General comments	**These are the most important comments that support and justify acceptance or refusal**. In this section of comments to authors, never state your opinion on whether the manuscript should or should not be accepted, not even the possibility of acceptance or rejection.
2.1. Originality	Assess originality and make a quick literature search in the topic and authors. Assess what has been published. This is the most common reason for refusal.
2.2 Validity	Check if the data are valid: sample, appropriate data collection and analysis, sound statistics. Avoid asking for more cases or analysis, unless it is possible. Are the results valid for other populations?
2.3 Relevance	State your opinion on whether the study is relevant and why. What is the importance of the findings in the specific area? How does the study suit the needs of our journals' readers?
2.4 Extras	Comment on other strengths (well written, significant sample size), weaknesses (inappropriate methodology, unreliable data analysis), severe mistakes or very important limitations, extension of the manuscript and its parts (appropriate, too short, too long).
3. Specific comments	List punctual formal and grammar mistakes, meaningless sentences, correction of tables and figures, specific questions about certain points (how participants were selected, ask more details about the methodology, ask for specific statistic methods, express doubts about data collection and analysis, and how measurements were taken). Check the references (if they correspond to the text where they are indicated and if they are in the correct order), at least some randomly. But do not exceed in detail here. What matters most is your opinion about the manuscript in the 'general comments' space.
CONFIDENTIAL COMMENTS TO EDITORS	Very important section. Do not skip it. Give your honest opinion on the manuscript. Here the reviewer can directly state to the editors his opinion on whether the manuscript should be accepted or refused. Be technical, but be aware that the manuscripts submitted to our journals usually have limitations. Avoid extreme rigidity! In your opinion, is publishing the article a priority? If approved, should there be an editorial about the article? State whether the manuscript requires minor, major or more extensive reviews. In case of rejection, can the manuscript be resubmitted after being fully rewritten (de novo submission)? Acceptance without any review is rare, but, if that is the case, justify!
